# A single-cell hematopoietic microenvironmental atlas reveals progressive maturation of bone marrow vascular niche

**DOI:** 10.1186/s13619-025-00265-7

**Published:** 2025-12-04

**Authors:** Lan-Yue Ma, Zhao-Hua Deng, Ke Bai, Yan-Mei Yu, Yin Huang, Rong-Rong Gao, Yu-Yan Li, Xiao-Ling Li, Jia-Xin Yang, Ya-Hai Shu, Jinjin Ma, Yang Liu, Qi Chen

**Affiliations:** 1 Center for Cell Lineage Atlas, Guangzhou Institutes of Biomedicine and Health, Chinese Academy of Sciences, Guangzhou, 510530 China; 2 University of Chinese Academy of Sciences, Beijing, 101408 China; 3 Guangdong Provincial Key Laboratory of Stem Cell and Regenerative Medicine, Guangdong-Hong Kong Joint Laboratory for Stem Cell and Regenerative Medicine, Joint School of Life Sciences, Guangzhou Institutes of Biomedicine and Health, Chinese Academy of Sciences, Guangzhou, 510530 China; 4 Guangzhou Medical University, Guangzhou, 511436 China; 5 The Innovation Centre of Ministry of Education for Development and Diseases, School of Medicine, South China University of Technology, Guangzhou, 510006 China; 6 The Institute of Future Health, South China of Technology, Guangzhou International Campus, Guangzhou, 511442 China

**Keywords:** Hematopoietic microenvironment, Niche atlas, scRNA-seq, Midkine

## Abstract

The interaction between hematopoietic stem and progenitor cell (HSPC) and its vascular niche is essential for supporting the homeostasis and reconstitution of hematopoietic system in adult bone marrow (BM), but a comprehensive atlas covering this HSPC-vascular niche crosstalk in multiple developmental stages and species is lacking. Here, we integrated single-cell transcriptomic data of HSPC and its vascular niches from fetal liver until aged BM, covering two species, two organs, and six developmental time points. Comparative analyses revealed dramatic differences in the gene expression, enriched pathway, and cell–cell communication between human fetal and adult BM. Notably, many of these differences were conserved between humans and mice. Multi-timepoint profiling of murine BM vascular niches revealed a stepwise maturation of gene expression, including critical niche factors such as SCF and CXCL12. Furthermore, analysis of this dynamic vascular niche atlas highlighted organ-specific features between fetal liver and BM niches, significant transcriptional changes in aged BM endothelial cells, and identified midkine as a previously unknown niche factor. Functional validation showed that transplanting HSPC into midkine knockout mice or treating with a midkine inhibitor (iMDK) enhanced hematopoietic reconstitution. In contrast, recombinant midkine suppressed HSPC differentiation. Together, our work presents a cross-species and multi-stage atlas of HSPC–vascular niche interactions, offering valuable insights into the dynamic changes of vascular niche through lifelong HSPC development and a platform to identify unknown niche factors.

**Graphical Abstract:**

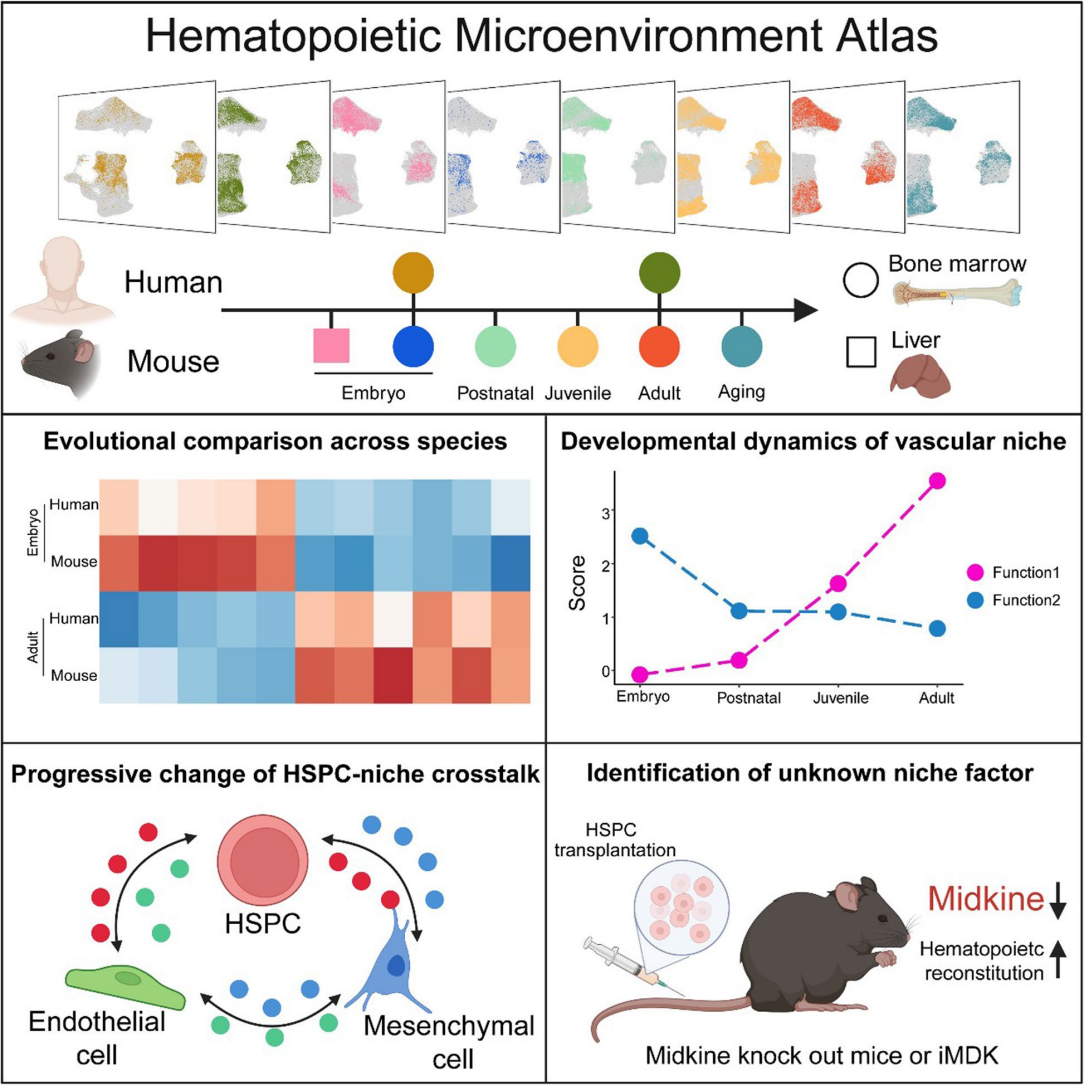

**Supplementary Information:**

The online version contains supplementary material available at 10.1186/s13619-025-00265-7.

## Background

Hematopoiesis is a fundamental biological process responsible for the continuous generation of blood and immune cells throughout an individual’s life (Kasbekar et al. 2023). These cells originate from hematopoietic stem and progenitor cells (HSPC), which primarily reside in the bone marrow (BM) of mammals (Comazzetto et al. 2021). The self-renewal, multipotent differentiation, and proliferation of HSPCs are tightly regulated by the bone marrow niche—a highly specialized microenvironment within the bone (Asada et al. 2017; Birbrair and Frenette 2016).

This niche comprises a complex network of cellular components, including bone marrow endothelial cells (BMEC), mesenchymal stromal cells (BMSC), osteolineage cells (OLC), nerves, adipocytes, and differentiated hematopoietic cells (Pinho and Frenette 2019). Among these, BMEC and BMSC are closely associated and together form the vascular niche, which plays a critical role in maintaining HSPC function (Tuckermann and Adams 2021). While various niche components influence HSPC via paracrine signaling and direct cell–cell interactions, mounting evidences indicate that most HSPC reside within 10 μm of the vascular niche, underscoring the pivotal role of BMEC and BMSC in HSPC regulation (Acar et al. 2015; Chen et al. 2019; Christodoulou et al. 2020).

HSPC ensures the lifelong production of blood cells and respond to physiological demands. However, the structure, composition, and function of the vascular niche evolve significantly from fetal development through adulthood and into old age (Kara et al. 2023; Langen et al. 2017). For instance, our previous research has highlighted the essential role of caveolin-1^+^ BMEC in fetal mouse BM (Liu et al. 2022), while aging is associated with the decline of type-H and arterial-like BMEC populations (Kusumbe et al. 2016). Despite these findings, most studies of HSPC-niche interactions have focused on steady-state conditions or responses to myeloablative injury in adult bone marrow (Hoggatt et al. 2016). Consequently, how the vascular niche develops and changes from the embryonic stage through adulthood—and how these changes affect its interaction with HSPC—remain poorly understood, particularly in humans, where bone marrow samples are scarce and experimental manipulation is limited.

In this study, we integrate single-cell RNA sequencing (scRNA-seq) datasets to construct a temporally dynamic atlas of the bone marrow vascular niche. This atlas enables us to compare fetal and adult bone marrow, examine cross-organ and cross-species differences, and identify previously uncharacterized niche factors.

## Results

### A single-cell atlas of HSPC and vascular niche from embryo to aging

**Fig. 1:**
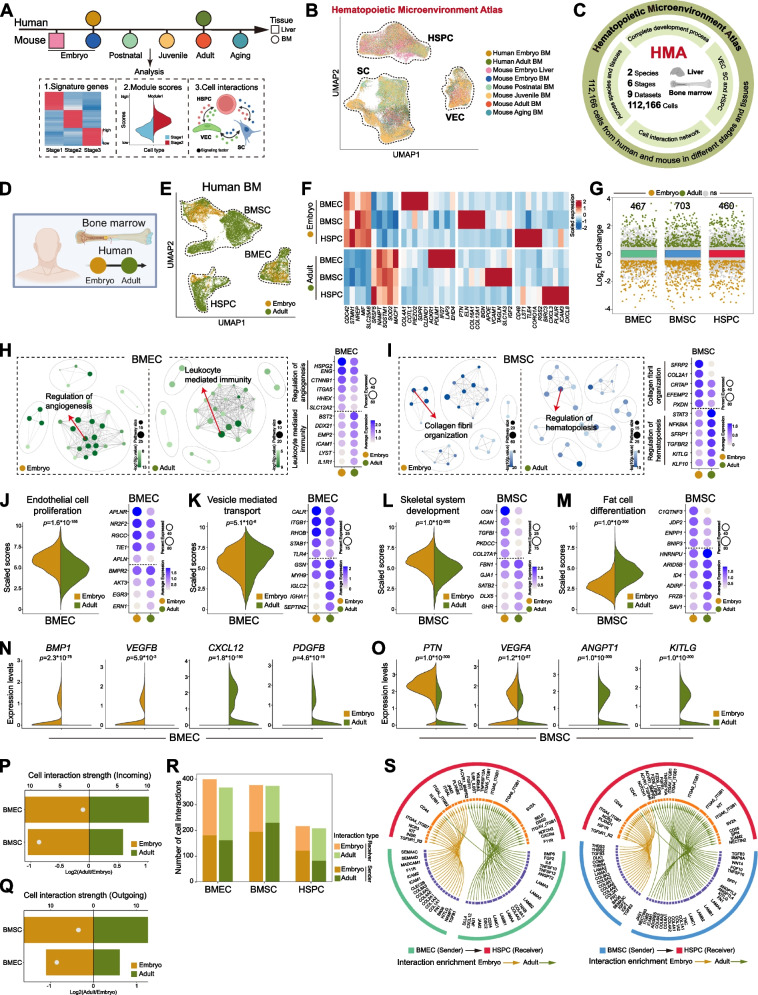
Generation of the hematopoietic microenvironment atlas and comparison of human embryonic and adult bone marrow. **A** Schematic representation of the hematopoietic microenvironment atlas (HMA) framework integrating multi-datasets and analysis. **B** UMAP visualization of single-cell transcriptomes cells based on developmental stages and species in hematopoietic microenvironment atlas (HMA). **C** Diagram highlighting key functional modules in the HMA. **D** Diagram showing data integration for human embryonic and adult BM. **E** UMAP plot showing cell types in human embryonic and adult BM. **F** Heatmap displaying different stage enriched genes across human BM cell types. **G** Quantitative comparison of differentially expressed genes (DEG) counts between human embryonic and adult BM. **H-I** Gene Ontology (GO) enrichment analysis of DEG and dot plot showing selective genes associated with GO in human bone marrow endothelial cell (BMEC) or bone marrow stromal cell (BMSC). **J-K** Violin plot showing endothelial proliferation activity and vesicle mediated transport scores and dot plot showing genes associated with human embryonic and adult BMEC. **L-M** Violin plot showing skeletal system development and fat cell differentiation scores and dot plot showing genes associated with human embryonic and adult BMSC. **N–O** Violin plots comparing niche factor expressions (*BMP1*, *VEGFB*, *CXCL12*, *PDGFB*, *PTN*, *VEGFA*, *ANPGT1*, *KITLG*) in human embryonic and adult BMEC (**N**) or BMSC (**O**). **P-Q** Quantification of incoming (**P**) or outgoing (**Q**) crosstalk strength in human embryonic and adult BMEC and BMSC. **R** Comparison of cell–cell communication pathway numbers in BMEC, BMSC, and HSPC in human embryonic and adult. **S** Enrichment signaling from BMEC or BMSC to HSPC in human embryonic and adult BM

To comprehensively assess the interactions between HSPC and their vascular niche across developmental stages, organs, and species, we integrated scRNA-seq datasets. These included vascular endothelial cells, perivascular mesenchymal cells, and HSPC from six developmental stages: fetal liver, fetal BM, neonatal BM, juvenile BM, adult BM, and aged BM (Fig. 1A-B). In total, this atlas includes 112,166 cells across two organs and two species, providing a robust framework for analyzing the vascular microenvironment and its regulatory role in hematopoiesis after dataset integration (Fig. 1C, Fig. S1A-F, Table S1). The following sections demonstrated how this atlas enabled comparative analyses and the identification of unknown therapeutic targets.

Firstly, we used the dataset in our atlas to compare the fetal and adult BM vascular niche in human (Fig. 1D-E and Fig. S1G-I). Comparative analysis identified human genes enriched within either fetal or adult BM, independent of cell type (Fig. 1F). This analysis also delineated cell type-enriched temporal signatures of BMEC and BMSC (Fig. 1F), revealing substantial transcriptional divergence between fetal and adult BM in human (Fig. 1G). Gene ontology (GO) analysis indicated that genes in BMEC were associated with angiogenesis and leukocyte-mediated immunity at corresponding stages (Fig. 1H), whereas BMSC exhibited differences in extracellular matrix organization and hematopoietic regulation (Fig. 1I). Pathway scoring revealed that fetal BMEC showed stronger VEGF signaling and proliferative capacity (Fig. 1J and Fig. S1J), while adult BMEC demonstrated better vehicle transmission ability (Fig. 1K and Fig. S1K). Fetal BMSC showed increased skeletal development potential (Fig. 1L and Fig. S1L), whereas adult BMSC exhibited adipogenic differentiation preference (Fig. 1M and Fig. S1M), which was consistent with reported murine properties (Shu et al. 2021).

One typical feature of the BM vascular niche was their ability to release microenvironmental factors (Mendez-Ferrer et al. 2020; Sanchez-Aguilera and Mendez-Ferrer 2017). A developmental shift in niche factor expression was detected in human BM, including key regulators such as stem cell factor (SCF, encoded by *KITLG*), CXCL12, ANGPT1, PTN, VEGF, and PDGF (Fig. 1N-O and Fig. S1N-O). Comparative analysis of vascular niche-HSPC interactions revealed an overall stronger interaction strength in fetal human BM (Fig. 1P-Q) with similar number of cell–cell interactions across fetal and adult human BM (Fig. 1R). However, HSPC received unidentical signals from BMEC and BMSC in the adult niche and their fetal counterparts (Fig. 1S), suggesting fetal and adult BM provided different microenvironment to support HSPC that was similarly detected in mice (Liu et al. 2022).

These data indicated that human fetal and adult bone marrow vascular niche cells differed substantially in transcriptional profiles, enriched signaling, niche factor secretion, and interaction networks with HSPC.

### Evolutionary conservation of vascular niche development between humans and mice

**Fig. 2:**
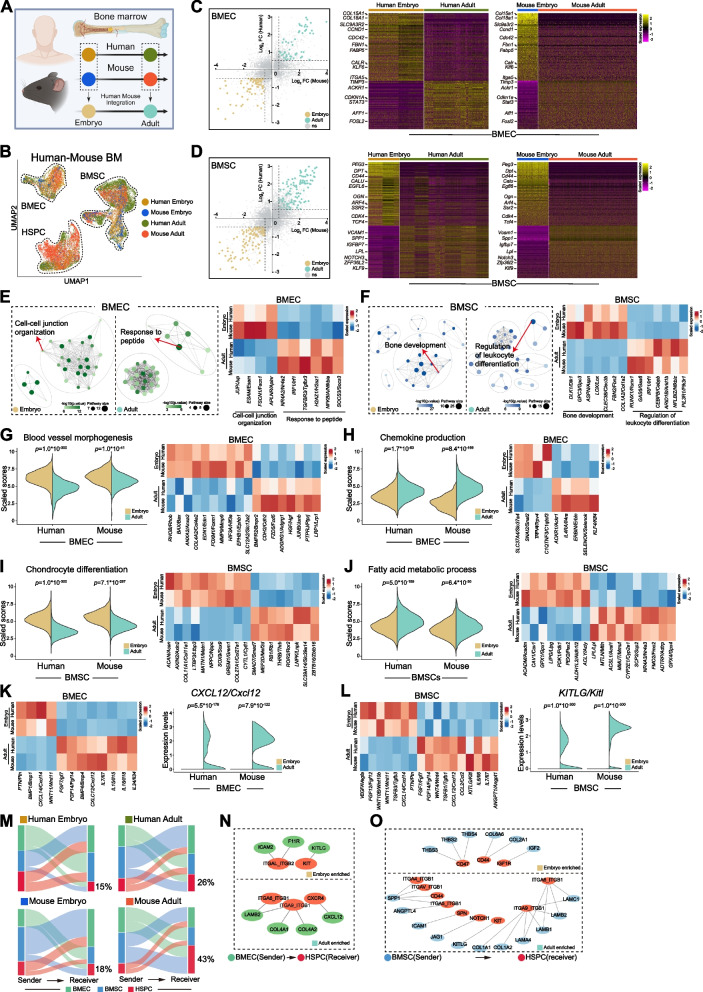
Evolutionarily conserved features between human and mice BM microenvironment. **A** Schematic of cross-species data integration strategy for embryonic and adult human/mouse BM. **B** UMAP plot showing cell types in human/mouse BM with embryonic and adult. **C-D** Conserved cross-species DEG between embryonic and adult stages, and heatmap of human-mouse conserved gene expression in BMEC or BMSC at each developmental stage. **E–F** GO enrichment analysis of cross-species conserved DEG and heatmap of representative GO-associated genes in embryonic and adult BMEC or BMSC. **G-H** Violin plots of blood vessel morphogenesis and chemokine production scores and heatmap of cross-species conserved and stage-specific genes in BMEC. **I-J** Violin plots of chondrocyte differentiation and fat acid metabolic process scores and heatmap of cross-species conserved and stage-specific genes in BMSC. **K-L** Heatmap and violin plot of species-conserved niche factor expressions between embryonic and adult in human/mouse BMEC or BMSC. **M** River plot showing percentage differences of HSPC as signal receivers between embryonic and adult stages in human and mouse. **N–O** Cross-species shared enriched from BMEC or BMSC to HSPC interactions in embryonic and adult stages

To determine whether the observed developmental shift was evolutionarily conserved across mammals, we conducted a cross-species comparison between human and mice (Fig. 2A-B and Fig. S2A-D). Differential expression analysis identified genes enriched in either fetal or adult stages within both BMEC and BMSC, revealing largely consistent temporal expression dynamics across species (Fig. 2C-D). GO analysis of these differentially-expressed genes (DEG) highlighted conserved biological processes, including cell–cell junction organization, response to peptide, bone development and regulation of leukocyte differentiation in BMEC or BMSC, with corresponding genes exhibiting similar expression trends in both humans and mice (Fig. 2E-F). Pathway scoring in BMEC revealed that genes associated with blood vessel morphogenesis and angiogenesis were predominantly enriched in embryo, whereas chemokine production were more prominent in adult (Fig. 2G-H and Fig. S2E-F), that was consistent with growth and maintenance function of BMEC in fetal and adult BM, respectively (Liu et al. 2022). In BMSC, fetal cells were enriched for chondrocyte differentiation, while adult cells exhibited increased activity in fatty acid metabolism (Fig. 2I-J and Fig. S2G-H). These pathway activities and gene expression changes demonstrated consistent evolutionary trends across species and were consistent with their known function.

Analysis of microenvironmental factor expression showed that both fetal and adult BMEC and BMSC exhibited stage-specific niche factor expression pattern that was conserved between humans and mice (Fig. 2K-L). Notably, the CXCL12 expression in BMEC and the SCF expression in BMSC significantly increased during adulthood in both species (Fig. 2K-L). Cell–cell interaction analysis demonstrated increased signaling percentage from BMEC and BMSC to HSPC in both human and mouse adult BM compared to embryonic stage (Fig. 2M), together with conserved vascular microenvironment-mediated signaling pathways, including SCF-KIT, CXCL12-CXCR4, IGF2-IGF1R and SPP1-integrin signaling in these two mammals (Fig. 2N-O and Fig. S2I).

Collectively, these findings demonstrated evolutionary conservation of vascular microenvironmental gene expression dynamics and their functional interactions with HSPC between humans and mice.

### Developmental dynamics of the murine bone marrow vascular microenvironment

**Fig. 3:**
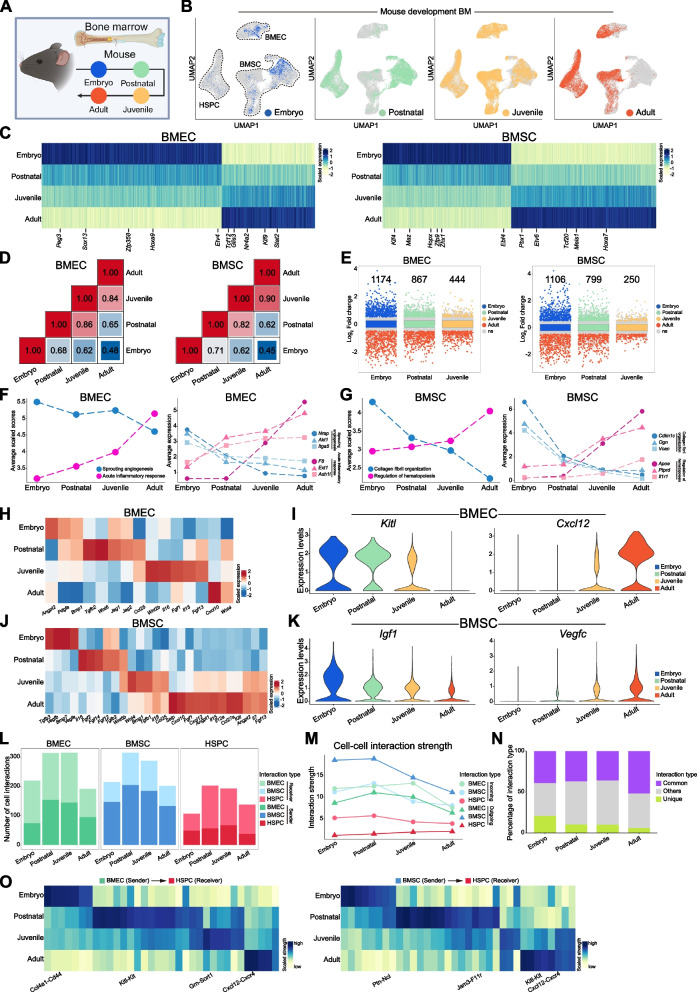
The dynamic changes of murine BM microenvironment during developmental maturation. **A** Schematic of developmental data integration from embryo, postnatal, juvenile and adult BM. **B** UMAP plot showing cell types in developmental mouse BM. **C** Dynamic gene expression trajectories in developmental BMEC and BMSC. **D** Heatmap of inter-stage correlations during BMEC and BMSC development. **E** Dynamic changes in DEG across embryonic, postnatal, and juvenile stages versus adult in BMEC and BMSC. **F** Developmental dynamics of sprouting angiogenesis and acute inflammation response scores and associated gene expression in mouse developmental BMEC. **G** Developmental dynamics of collagen fibril organization and regulation of hematopoiesis scores and associated gene expression in mouse developmental BMSC. **H-I** Heatmap of niche factor expression and violin plots of *Kitl* and *Cxcl12* expression levels across developmental BMEC. **J-K** Heatmap of niche factor expression and violin plots of *Igf1* and *Vegfc* expression levels across developmental BMSC. **L** Number of cell–cell communication pathways in BMEC, BMSC, and HSPC across developmental BM. **M** Quantification of incoming and outgoing crosstalk strength across developmental BM. **N** Percentage of interaction types across developmental BM. **O** Heatmap of BMEC or BMSC as sender and HSPC as receiver signaling strength across developmental stages of BM

Because of the evolutionary similarities between human and murine BM microenvironments and the availability of samples from the BM of mice, we analyzed the developmental dynamics of BMEC and BMSC across fetal, postnatal, juvenile, and adult stages from the atlas (Fig. 3A-B and Fig. S3A-C). Multiple developmental time point analysis identified a list of genes showing temporal transition in the gene expression level from embryo to adult (Fig. 3C). Pearson correlation analysis revealed a gradual transcriptional transition in BMEC and BMSC from embryo through postnatal/juvenile until adulthood (Fig. 3D). Similarly, the number of DEG, comparing adult BMEC or BMSC to other developmental stages, decreased during the maturation of these cells (Fig. 3E). Representative gene sets could be selected to assess the developmental dynamics of key pathway, including angiogenesis in BMEC and hematopoiesis regulation in BMSC, further supported this step-by-step maturation pattern (Fig. 3F-G and Fig. S3D-E).

Analyzing the developmental dynamics of vascular niche factors revealed gradual downregulation of SCF and Pdgfb, alongside upregulation of CXCL12 and Selp in BMEC (Fig. 3H-I and Fig. S3F). Meanwhile, BMSC showed decreasing expression of IGF1 and Ptn together with upregulating Vegfc and Spp1 (Fig. 3J-K and Fig. S3G). Notably, cell–cell interaction analysis revealed peak interaction number among BMEC, BMSC and HSPC at the postnatal and juvenile stages, rather than embryonic or adult periods (Fig. 3L and Fig. S3H). Crosstalk strengths were relatively stable in HSPC, but the interaction strengths were gradually reduced after postnatal in BMSC and BMEC (Fig. 3M). Among these interactions, 30–50% were conserved across all timepoints, while 10–20% were developmental stage-specific (Fig. 3N), including relative enrichment of individual pathway with stronger signaling strength at each developmental stage (Fig. 3O).

These findings indicated that while BMEC and BMSC underwent gradual maturation in transcription and niche factor secretion, their putative interactions with HSPC were potentially more active during postnatal development.

### Organ-specific vascular microenvironments in fetal bone marrow and liver

**Fig. 4:**
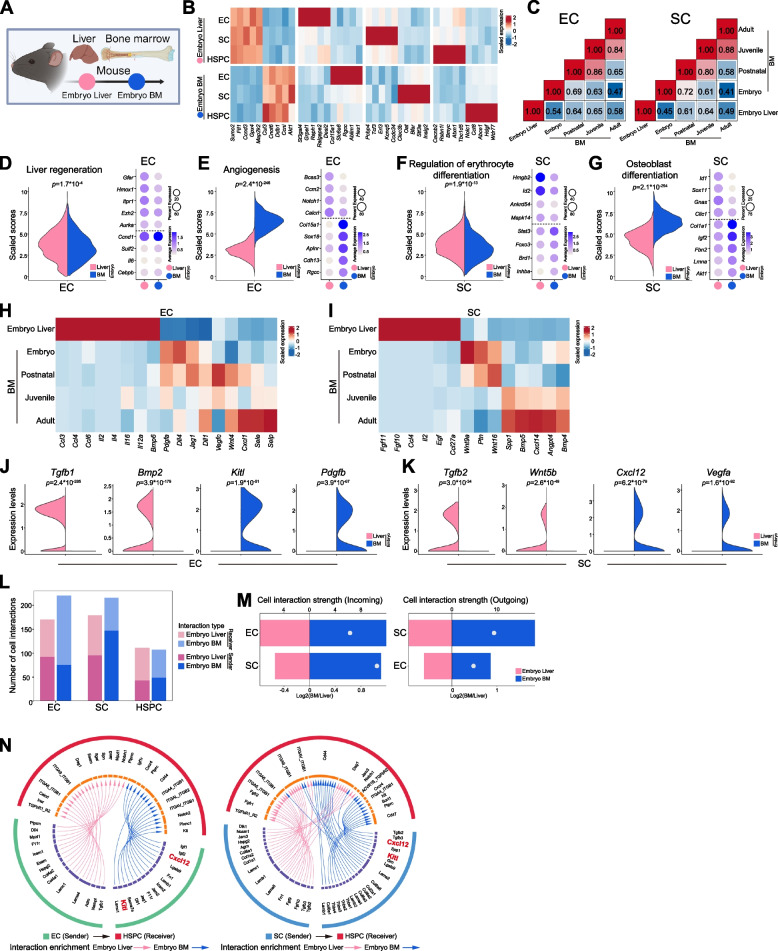
scRNA-seq comparison of murine fetal liver and BM. **A** Diagram showing data integration for embryonic liver and BM. **B** Heatmap displaying tissue-specific expressed genes across cell types. **C** Heatmaps showing correlation between liver and developmental BM in Endothelial cell (EC) and Stromal cell (SC). **D-E** Violin plot showing liver regeneration and angiogenesis scores and dot plot showing genes associated with embryonic liver and BM in EC. **F-G** Violin plot showing regulation of erythrocyte differentiation and osteoblast differentiation scores and dot plot showing genes associated with embryonic liver and BM in SC. **H-I** Heatmap of niche factor expression in embryonic liver and developmental BM in EC or SC. **J-K** Violin plots comparing niche factor expressions (*Tgfb1*, *Bmp2*, *Kitl*, *Pdgfb*, *Tgfb2*, *Wnt5b*, *Cxcl12*, *Vegfa*) between embryonic liver and BM in EC or SC. **L** Comparison of cell–cell communication pathway numbers of EC, SC, and HSPC in embryonic liver and BM. **M** Quantification of incoming and outgoing crosstalk strength between embryonic liver and BM in EC and SC. **N** Enrichment signaling from EC or SC to HSPC in embryonic liver and BM

Before HSPC engraftment into the fetal BM at E16.5 to interact with BM vascular niche (Lee et al. 2021; Liu et al. 2022), the hepatic vascular microenvironments promote HSPC expansion (Khan et al. 2016). However, the vascular niche comparison between fetal BM and fetal liver was very limited. Therefore, we extracted these datasets from our atlas (Fig. 4A and Fig. S4A-D), identifying organ-specific genes including Sumo2 or Cul3 that were highly enriched in the fetal liver or bone marrow, respectively (Fig. 4B). Pearson’s coefficient correlation analysis revealed that the endothelial and mesenchymal cells in fetal liver were dramatically different from the corresponding cells across all stages in BM (Fig. 4C). BMEC and BMSC subpopulation analysis revealed that the endothelial and mesenchymal cells in fetal liver were not identical to any subtype of corresponding fetal BM cells (Fig. S4E). These data suggested substantial organ-specificity in the vascular microenvironments, with fetal liver niche cells showing limited resemblance to any subtype of BM vascular niche. DEG analysis further underscored microenvironmental divergence, identifying numerous genes differentially expressed between fetal liver and BM in endothelial and mesenchymal compartments (Fig. S4F). GO analysis linked hepatic endothelial DEG to liver regeneration (Fig. 4D-E and Fig. S4G-H). While liver mesenchymal DEG were associated with erythrocyte differentiation, bone marrow mesenchymal DEG showed preference to osteoblast differentiation that was consistent with their organotypic function (Fig. 4F-G and Fig. S4I-J).

Niche factor analysis revealed that fetal liver endothelial and mesenchymal cells showed higher expression of some growth factor or chemokine in the CCL family, FGF family and interleukin family (Fig. 4H-I). Bone marrow expressed higher levels of SCF (*Kitl*), CXCL12, and pleiotrophin, even though the fetal liver was enriched for Tgfb1 and Tgfb2, which remained minimally expressed in BM (Fig. 4J-K). Cell–cell interaction analysis showed that fetal liver had fewer and weaker interactions among vascular endothelial cells, mesenchymal cells compared to fetal BM (Fig. 4L-M). In detailed signaling, the fetal liver and fetal BM displayed enrichment of individual signaling in each organ, but the key SCF and CXCL12 signaling was highly enriched in fetal BM (Fig. 4N), suggesting a superior ability of the BM niche to attract and retain HSPC.

These findings revealed fundamental differences in the vascular niche between fetal liver and bone marrow, which may underlie the eventual preference of HSPC for bone marrow residency.

### Aging-associated endothelial changes in the bone marrow niche

**Fig. 5:**
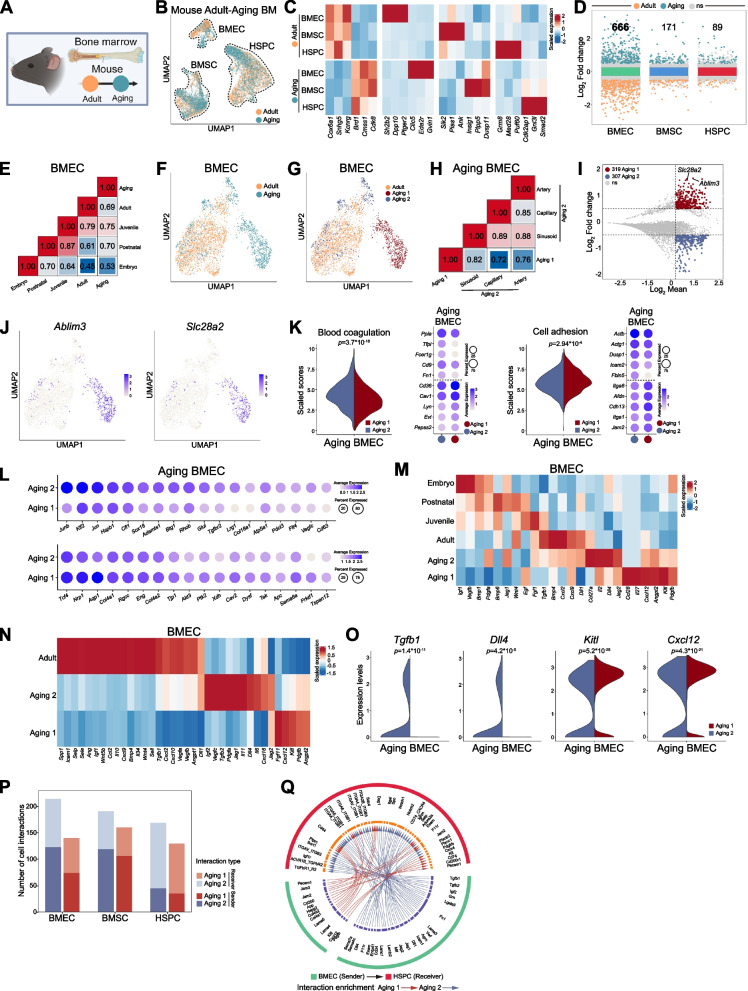
Aging-associated transcriptional changes in BM vascular microenvironment. **A** Diagram showing data integration for adult and aging BM. **B** UMAP plot showing cell types in adult and aging BM. **C** Heatmap displaying stage-specific expressed genes across BM cell types. **D** Number of DEG across cell types between adult and aging BM. **E** Heatmap showing correlation between aging and developmental BMEC. **F** UMAP plot showing BMEC from adult and aging. **G** UMAP plot of BMEC subpopulations in adult, aging 1 and aging 2. **H** Heatmap of correlation between aging 1 and aging 2 subclusters in BMEC. **I** MA plot of DEG between aging 1 and aging 2 in aging BMEC. **J** UMAP plots showing selected aging 1 signature genes (*Ablim3*, *Slc28a2*) in BMEC. **K** Violin plots showing blood coagulation and cell adhesion scores and dot plot showing genes associated with aging 1 and aging 2 in aging BMEC. **L** Dot plot of aging 1 and aging 2 BMEC signature genes associated with angiogenesis, endothelial cell proliferation, vascular tube morphogenesis, and VEGF signaling pathways. **M** Heatmap of niche factor expressions in developmental BM, aging 1 and aging 2 in BMEC. **N** Heatmap of niche factor expressions in adult, aging 1 and aging 2 in BMEC. **O** Violin plots comparing niche factor expressions (*Tgfb1*, *Dll4*, *Kitl*, *Cxcl12*) in aging 1 and aging 2. **P** Number of cell–cell communication pathways across different cell types, BMEC were stratified into aging 1/2 subclusters, while BMSC and HSPC remained unstratified. **Q** Enrichment signaling from BMEC to HSPC in aging 1 and aging 2 BM

Next, we extended our analysis to compare the adult and aged BM microenvironments (Fig. 5A-B and Fig. S5A-C). Our analysis identified expression of genes which were mostly associated with aging 3 cell types and enriched in specific cell type (Fig. 5C). However, aging resulted in greater impact on gene expression in vascular endothelial cells than in mesenchymal cells or HSPC that was similarly detected comparing adult and aging BM cells in DEG numbers and Pearson’s coefficient correlation analysis (Fig. 5D-E and Fig. S5D), which triggered us to focus on BMEC comparison between adult and aged BM.

Clustering of adult and aged endothelial subpopulations revealed a unique endothelial subtype enriched in aged bone marrow (defined as aging 1), which was distinct from arterial, capillary, and sinusoidal endothelial cells in the aged bone marrow (Fig. 5F-G and Fig. S5E-G). This aging 1 subpopulation showed higher CD38 expression, which was low in postnatal and young adult BMEC (Fig. S5H-I). This aging 1 subpopulation displayed transcriptional profiles that differed from embryonic, neonatal, juvenile, and adult BMEC (Fig. S5J), as well as from other aged BMEC subtypes (Fig. 5H). DEG analysis identified more than 300 genes that distinguished aging 1 with other aged endothelial subtypes (Fig. 5I-J), with GO analysis linking them to weaker association with blood coagulation and permeability (Fig. 5K and Fig. S5K). Genes associated with endothelial function, including Klf2, Sox18, Nrp1, Lrg1, were changed in the aging 1 cluster (Fig. 5L). We noted that some microenvironmental factors were highly expressed in the aging 1, including *Kitl* and *Cxcl12* (Fig. 5M-O). However, aging 1 showed overall weaker expression of a substantial number of other microenvironmental factors (Fig. 5N-O). Therefore, although there was no dramatic change of cell–cell interaction numbers between adult and aging BM (Fig. S5L-M), the aging 1 showed reduced ability to interact with other cell types (Fig. 5P-Q).

These data indicated that aging dramatically modified the transcriptome of BMEC.

### Midkine was an uncharacterized microenvironmental factor

**Fig. 6:**
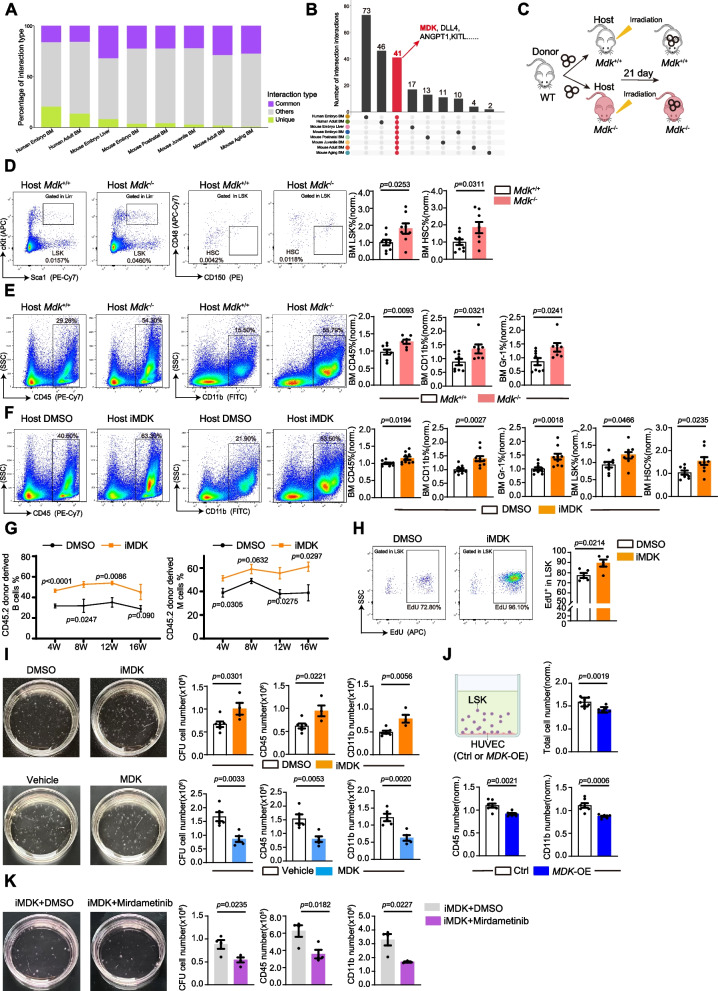
Midkine was an uncharacterized microenvironmental factor. **A** Quantifying the type of cell–cell interaction across all stages in the atlas. **B** Upset plot showing HSPC as receiver between common and unique cell–cell interactions across all stages in the atlas. **C** Diagram depicting treatment of *Mdk*^+/+^and *Mdk*^−/−^ mouse after lethal irradiation and transplantation as well as corresponding analyzing time point. **D** Representative FACS contour plot and quantification about the percentage of Lin^−^ c-Kit^+^ sca1^+^(LSK cell, *Mdk*^+/+^ = 9, *Mdk*^−/−^ = 8), hematopoietic stem cell (HSC, *Mdk*^+/+^ = 9, *Mdk*^−/−^ = 8) 21 days after transplantation. Error bars, mean ± SEM. *p* values, t- test. **E** Representative FACS contour plot of CD45 (*Mdk*^+/+^ = 8, *Mdk*^−/−^ = 7), CD11b (*Mdk*^+/+^ = 8, *Mdk*^−/−^ = 7) and quantification about the percentage of CD45, CD11b and Gr-1 (*Mdk*^+/+^ = 8, *Mdk*^−/−^ = 7) at 21 days after transplantation. Error bars, mean ± SEM. *p* values, t- test. **F** Representative FACS contour plot of CD45 (DMSO = 9, iMDK = 10), CD11b (DMSO = 9, iMDK = 10) and quantification about the percentage of CD45, CD11b, Gr-1(DMSO = 9, iMDK = 10) LSK (DMSO = 8, iMDK = 9), HSC (DMSO = 8, iMDK = 9) after transplantation. Error bars, mean ± SEM. *p* values, t- test. **G** Competitive repopulation assay donated by DMSO (CD45.2, *n* = 4) or iMDK (CD45.2, *n* = 4) mice bone marrow cells which were approximately 1:1.5 mixed with CD45.1 donor cells and transplanted into lethally irradiated CD45.1 host mice. Peripheral blood was analyzed at indicted time points. Error bars, mean ± SEM. *p* values, t- test. **H** FACS plot quantifying the percentage of EdU^+^ cell percentage in LSK in both DMSO (*n* = 5) and iMDK (*n* = 5) mice after drug treatment. Error bars, mean ± SEM. *p* values, t- test. **I** Representative images (dish at day 8 after seeding) and quantification of CFU total cell number, CD45 number or CD11b number derived from 20,000 Lin^−^ cell isolated from WT mice. DMSO or iMDK was added to MethoCult medium (DMSO = 6, iMDK = 4, Vehicle = 5, MDK = 5). Error bars, mean ± SEM. *p* values, t- test. **J** Control (*n* = 7) or *MDK*(*n* = 7) overexpression of HUVEC influence myeloid cell production when HUVEC were co-cultured with lineage-negative HSPC. Error bars, mean ± SEM. *p* values, t- test. **K** Representative images (dish at day 8 after seeding) and quantification of CFU total cell number (DMSO + iMDK = 4, iMDK + Mirdametinib = 4), CD45 number (DMSO + iMDK = 4, iMDK + Mirdametinib = 4) or CD11b number (DMSO + iMDK = 4, iMDK + Mirdametinib = 3) derived from 20,000 Lin^−^ cell isolated from WT mice. DMSO + iMDK or iMDK + Mirdametinib was added to MethoCult medium. Error bars, mean ± SEM. *p* values, t- test

Our previous analysis revealed dynamic expression patterns of vascular niche factors across different developmental stages in the BM. To identify a common microenvironmental factor that persistently existed during vascular niche-HSPC crosstalk, we screened our atlas discovering midkine, a homolog of niche factor pleiotrophin, constantly formed putative communications with HSPC from fetal liver to aged bone marrow (Fig. 6A-B and Fig. S6A-B). However, the function of midkine to regulate HSPC was not validated in genetically-modified mice in vivo. Therefore, we generated a midkine knockout mice (*Mdk* KO) which exhibited normal hematopoiesis under steady-state condition (Fig. S6C-D). However, wildtype HSPC transplantation into *Mdk* KO recipient mice resulted in increased LSK cell and leukocyte percentage (Fig. 6C-E), suggesting absence of midkine in niche may promote hematopoietic reconstitution after transplantation.

This triggered us to test whether midkine was a potential target to enhance hematopoietic transplantation efficiency. Pharmacological inhibition of midkine synthesis using iMDK, an antagonist that inhibit midkine generation (Khan et al. 2016), elevated LSK and leukocyte cell percentage after transplantation (Fig. 6F and Fig. S6E). This evidence suggested that iMDK, a reagent without apparent harmful effect in normal cells and mice (Ishida et al. 2015), had the potential to promote bone marrow transplantation. CD45.1/CD45.2 competitive transplantation assay confirmed that midkine inhibition enhanced the proportion of HSPC in BM (Fig. 6G), which was possibly because iMDK promoted the proliferative EdU^+^ HSPC (Fig. 6H). Similar to BM, iMDK treatment influenced hematopoiesis in fetal liver (Fig. S6F-H).

To confirm the inhibitory effect of midkine, we performed the colony forming unit (CFU) assay in which iMDK increased colony proliferation and blood cell number, while supplementing recombinant midkine suppressed the number of hematopoietic cells (Fig. 6I). Moreover, co-culture of HSPC with endothelial cells overexpressing midkine resulted in reduced leukocyte generation ability in vitro (Fig. 6J and Fig. S6I). Finally, when iMDK was added together with Mirdametinib, an inhibitor of MEK/ERK signaling, the number of hematopoietic cells significantly decreased in CFU assay (Fig. 6K and Fig. S6J), suggesting MEK/ERK was associated with midkine signaling.

These findings suggested our atlas could be utilized to identify midkine as a putative microenvironmental factor that influenced HSPC proliferation and inhibiting midkine signaling as a potential approach to enhance hematopoietic reconstitution after transplantation.

## Discussion

This work establishes a vascular niche atlas that systematically compares developmental and adult BM, revealing evolutionarily conserved inter-species features between murine and human BM. The dataset spans embryonic, postnatal, juvenile, and adult vascular niches, facilitating comparisons between fetal liver and fetal BM microenvironments, and enabling the identification of unique transcriptomic endothelial profile in aged BM. Multi-timepoint analysis uncovers midkine as a previously uncharacterized microenvironmental factor that constrains excessive HSPC proliferation. This finding leads to the identification of iMDK as a pharmacological agent with the potential to enhance hematopoietic reconstitution following HSPC transplantation.

During mammalian hematopoietic development, the BM microenvironment of HSPC undergoes significant changes (Deng et al. 2023; Kasbekar et al. 2023). In the fetal BM, arterial blood vessels mediate initial HSPC engraftment, while Lepr⁺ BMSC are largely absent (Liu et al. 2022). In the postnatal and juvenile stages, BMEC support HSPC maintenance via membrane-bound SCF, and Lepr⁺ BMSC begin contributing to myeloid and erythroid lineage expansion (Kara et al. 2023). In adulthood, Lepr⁺ BMSC become more prevalent, promoting a shift in HSPC from a proliferative, oxidative metabolic state to a quiescent state with enhanced immunomodulatory function. Simultaneously, endothelial SCF expression declines, and HSPC transition to specialized, quiescence-promoting niches (Kara et al. 2023; Kasbekar et al. 2023; Takizawa et al. 2011). Adult HSPC relies more on glycolytic metabolism to maintain quiescence, supporting long-term preservation and adaptive responses to stress through crosstalk with the BM vascular niche (Kasbekar et al. 2023). The dynamic transcriptional changes observed in our study align closely with these known developmental transitions of the vascular niche. Notably, our atlas includes multiple datasets with noticeable variation because of batch effect or platform heterogeneity before data integration. However, SCTransform effectively integrate these datasets generating comprehensive and convincing atlas.

Prior to engraftment into fetal BM, the fetal liver serves as the primary hematopoietic organ, supporting HSPC expansion and erythro-myeloid differentiation through a specialized microenvironment, including SCF⁺DLK1⁺ stromal cells (Lewis et al. 2021). However, this support is transient and developmentally limited. For instance, fetal liver-derived B lymphocytes develop independently of IL-7Rα signaling, but this characteristic disappears within one to two weeks after birth (Tsuneto et al. 2013). HSPC from the fetal liver also shows weaker competitive potential when transplanted into adult bone marrow (Kikuchi and Kondo 2006). Single-cell transcriptomic data suggest that fetal liver HSPC, particularly in early gestation, exhibits low stemness, genomic instability, and abnormal interferon signaling, which may contribute to the fetal origin of childhood leukemia (Xie et al. 2024). Additionally, their reliance on oxidative phosphorylation renders them metabolically inefficient in the hypoxic adult BM, limiting post-transplant reconstitution capacity (Lewis et al. 2021; Xie et al. 2024). Although the fetal liver microenvironment supports HSPC during embryogenesis, our analysis suggests that it is fundamentally distinct from the BM niche, with organ-specific characteristics reminiscent of recent studies uncovering endothelial cell heterogeneity across tissues (Augustin and Koh 2017; Gomez-Salinero et al. 2021; Petrova and Koh 2018; Trimm and Red-Horse 2023).

The aging of HSPC is driven not only by intrinsic mechanisms but also by progressive remodeling of the bone marrow microenvironment. In aged BM, BMSC exhibit a shift from osteogenic to adipogenic differentiation potential, impairing the secretion of niche factors essential for maintaining HSPC quiescence (Ho and Mendez-Ferrer 2020). Aging is also associated with dysregulated sympathetic signaling, with reduced β3-adrenergic receptor activity promoting vasoconstriction and excessive β2-adrenergic signaling contributing to myeloid and megakaryocytic bias in HSPC fate (Gadomski et al. 2022; Maryanovich et al. 2018). Transcriptomic profiling of aged BMEC reveals a distinct Aging 1, consistent with vascular decline in aged marrow (Kusumbe et al. 2016). Furthermore, aged bone marrow exhibits upregulation of pro-inflammatory cytokines (e.g., IL-1β, IL-6, CXCL9/10) and activation of the complement system, reflecting a chronic low-grade inflammatory state (Helbling et al. 2019). Concurrent downregulation of extracellular matrix genes, such as collagens and matrix-remodeling enzymes, further degrades bone marrow integrity (Helbling et al. 2019). Reduced IGF1 levels have also been shown to accelerate HSPC aging and increase susceptibility to myeloid malignancies (Young et al. 2021). These microenvironmental changes are tightly associated with HSPC dysfunction and point to the potential of niche-targeted therapies to combat age-related hematopoietic decline.

Midkine is a heparin-binding growth factor composed of N- and C-terminal domains flanking a hinge region, which mediates interactions with heparan and chondroitin sulfate proteoglycans (Muramatsu 2014). It is abundantly expressed in the embryonic central nervous system, where it promotes neurogenesis, neuronal survival, and oligodendrocyte precursor differentiation (Neumaier et al. 2023). Under pathological conditions, midkine expression is upregulated by hypoxia-inducible factor-1α (HIF-1α) and NF-κB, linking it to both hypoxia and inflammation (Neumaier et al. 2023). In cancer biology, midkine has been implicated in tumor progression in glioblastoma and neuroblastoma through ALK/NF-κB and Notch2/Hes1 signaling pathways (Muramatsu and Kadomatsu 2014; Neumaier et al. 2023). The midkine inhibitor iMDK has shown anti-tumor efficacy in non-small cell lung cancer by blocking the PI3K-AKT pathway (Ishida et al. 2015). Additionally, iMDK induces apoptosis in primary effusion lymphoma cells by inhibiting CDK1 phosphorylation (Ueno et al. 2022). Our findings extend the biological relevance of midkine to hematopoiesis. While midkine knockout mice exhibit no overt hematopoietic defects under homeostatic conditions, they show enhanced hematopoietic reconstitution after HSPC transplantation. Similarly, pharmacological inhibition with iMDK increases the number of LSK and leukocyte populations post-transplantation, with no apparent toxicity in normal cells suggesting iMDK as potential approach to enhance transplantation efficiency. The proliferation of HSPC is properly balanced in both physiological condition and hematopoietic reconstitution. Even though HSPC displayed robust ability to replenish the bone marrow after injury (Cheshier et al. 1999), unlimited HSPC proliferation is harmful for hematopoietic system and the health. Unnecessary HSPC proliferation leads to DNA damage accumulation, which induced malfunction of this important cell type (Tasdogan et al. 2017). Continuous replenishment of the immune system and blood cells places a high demand on HSPC, and sustained high levels of stress on these cells can lead to an exhausted HSPC pool (Zhang et al. 2016), reducing their ability to respond to additional emergent condition. During continuous proliferation, HSPC is easier to become malignant (Carroll and St Clair 2018). A hallmark of leukemic hematopoiesis is unlimited generation of hematopoietic cells (Passegue et al. 2003). Therefore, the essential function of bone marrow niche is to properly control the proliferation of HSPC rather than enhancing its proliferation without any hurdle (Pinho and Frenette 2019). That may explain, at least partially, why the bone marrow niche needs inhibitory factor for HSPC proliferation.

Taken together, our vascular niche atlas provides a powerful resource to decipher the dynamic changes in the bone marrow microenvironment and its interaction with HSPC. Through this approach, we propose the inhibition of midkine as a promising strategy to enhance bone marrow transplantation.

## Materials and methods

### Animal experiments

C57BL/6 J male mice were used for all analysis of wild-type mice. Mice were sacrificed between 8 and 10 am. Animals were housed in the animal facility of Guangzhou Institutes of Biomedicine and Health. Animal experiments were performed according to the institutional guidelines and laws, following the protocols (2,021,059) approved by local animal ethics committees.

*Mdk* knockout mice (C57BL/6JGpt-*Mdk*^em5Cd3216^/Gpt) were generated by GemPharmatech (Strain NO. T012092, Nanjing, China). All animals were routinely genotyped using respective PCR protocols. Protocols and primer sequences can be provided upon request. iMDK (MCE, HY-110171; 12.8 mg/kg; in DMSO) was administrated by intraperitoneal injection (i.p.) for 4 times after irradiation. In the fourth time, mice were analyzed in 12 h after pharmacological treatment. 10 mg/kg EdU (K1078 EdU Flow Cytometry Assay Kits (Cy5), APExBIO, K1078) was administrated by intraperitoneal injection (i.p.) in 4 h after iMDK treatment. EdU analysis was performed following manufacture’s instruction.

### Irradiation and transplantation

Mice were exposed to a lethal dosage of irradiation followed by bone marrow cells transplanted at 4 to 6 h after irradiation. For competitive repopulating assays, CD45.1 host mice were lethally irradiated and transplanted with approximate 2 $\times$ 10^6^ donor-derived (CD45.2 treated by DMSO or iMDK) BM cells together with approximate 3 $\times$ 10^6^ host-derived (CD45.1 background) bone marrow cells. Peripheral blood from tail vein were analyzed by FACS to determine chimaeras level every 4 weeks.

### Flow cytometry

Bones were dissected and crashed by pestle for more than 3 times before cells were collected in 2% FCS-PBS solution. The tissue was immersed in 3 ml dissociation solution (2% FCS-PBS solution with approximate 145U/ml type 4 Gibco collagenase) and incubated at 37 °C for 30 min. Samples were filtered using 100 μm Nylon cell strainer (Biosharp, BS-100-XBS) to get single cell suspensions.

Cells were washed by 2% FCS-PBS solution and then incubated with primary antibodies on ice for 30 min. Cells were washed again and resuspended in 2% FCS-PBS for flow cytometry. The following primary antibodies were used in this study: mouse lineage cocktail (Biolegend, 133,307), Sca1-PE/Cy7 (BD, 558,162), cKit-APC (Biolegend, 105,812), CD150-PE (Biolegend, 115,904), CD48-APC/Cy7 (Biolegend, 103,432), CD45.1 (Biolegend, 110,706), CD45.2 (Biolegend, 109,808), CD45-PE/Cy7 (Biolegend, 103,114), CD11b-FITC (Biolegend, 101,206), Gr-1-APC (Biolegend, 108,412). Cells were washed times by 2% FCS-PBS solution and incubated with secondary antibodies for 30 min if necessary. Cells were washed times and used for flow cytometry. Cell sorting was performed on a FACS AriaIIu cell sorter (BD Biosciences, LSR Fortessa SORP).

For intracellular staining of EdU by FACS after surface staining of LSK cells, EdU staining was performed following the manufacturer’s instructions (K1078 EdU Flow Cytometry Assay Kits (Cy5), APExBIO, K1078). Next, cells were stained with mouse lineage cocktail (Biolegend, 133,307), Sca1-PE/Cy7 (BD, 558,162), cKit-APC (Biolegend, 105,812) at room temperature for 20 min. Cells were washed times and used for flow cytometry. Cell sorting was performed on a FACS AriaIIu cell sorter (BD Biosciences, LSR Fortessa SORP).

### Methylcellulose assay

Approximate 2 $\times$ 10^4^ Lin^−^ cells from wildtype mice were sorted by FACS and cultured in MethoCultTm medium (GFM3434, Stem cell technologies) in 35 mm dish. Lin^−^ cells were sorted into 2%FCS-PBS, with iMDK (150 nM in DMSO), MDK recombinant protein (MCE, HY-P73295, 300 ng/ml in ddH_2_O), Mirdametinib (MCE, HY-10254, 50 nM in DMSO) and control vehicle. Drugs or control vehicle were mixed with MethoCultTm medium during the seeding of Lin^−^ cells. Cells and medium were incubated in 37 °C 5%CO2 cell incubator. 8 days after incubation, CFU number was counted. After counting of CFUs, cells were resuspended and collected into 2% FCS PBS solution and filtered with 70 μm filter for FACS staining analysis and cell counting.

### Cell culture

The human umbilical vein endothelial cells (HUVECs) were cultured in ECM medium (ScienCell, 1001) with extra Endothelial Cell Growth Supplement (EGCS) (ScienCell, 1052) and grown in 5% CO_2_ at 37 °C. HEK293T/17 cells were seeded in DMEM supplement with 10% FCS and P/S.

The HEK293T/17 cells were transiently co-transfection with lentivirus using pLL3.7-GFP/PAX2/pVSVG or *hMDK*-overexpression/PAX2/pVSVG to produce corresponding virus for infection. Every batch of concentrated virus was tested by direct infection of HEK293T/17 cells. For infection of HUVEC cells, 10 µg/ml polybrene and 20 μl lentivirus (pLL3.7-GFP, or *hMDK*-OE) was added to the culture medium before infection. 3 days after infection, HUVEC cells were collected in RLT lysis buffer for RNA extraction.

### HSPC and HUVEC co-culture assay

Bone marrow cells was prepared as described in the flow cytometry method above. The following primary antibodies were used in this study: mouse lineage cocktail (Biolegend, 133,307), Sca1-PE/Cy7 (BD, 558,162), cKit-APC (Biolegend, 105,812). The LSK cells (Lin^−^ Sca1^+^ cKit^+^) sorting was performed on a FACS AriaIIu cell sorter (BD Biosciences, FACS Aria IIU) using a 85 µm nozzle. LSK cells (1 $\times$ 10^4^/700 μl) co-cultured with HUVECs for 8 days. After incubation, LSK cells number was counted. After counting of LSK cells, cells were resuspended and collected into 2% FCS PBS solution and analyzed for FACS staining.

### RNA extraction and quantitative PCR

RNA was extracted using AxyPrep Multisource Total RNA Miniprep Kit (Axygen, AP-MN-MS-RNA-250G) and cDNA was generated with HiScript II Q RT SuperMix for qPCR (Vazyme, R222-01). Quantitative reverse transcription PCR (qRT–PCR) was performed using Real-Time PCR system (Bio-Rad, America) and TB Green® Premix Ex Taq™ II (TaKaRa, RR820B). The primers were designed and synthesized by GenwWiz (Guangzhou, China). GAPDH was used as internal controls, and all reactions were performed in triplicate.

### Cryosectioning, immunohistochemistry and confocal imaging

Femoral bones were dissected and placed in ice- cold 4% paraformaldehyde (PFA-PBS) solution and fixed overnight. Bones were placed in 0.5 M EDTA (PH 8.0), dehydrated in 20% sucrose-1% polyvinylpyrrolidone PBS solution, and embedded in PBS containing 20% sucrose, 8% gelatin, and 1% polyvinylpyrrolidone for storage at—80 °C. Cryosectioning was performed on a Leica CM3050S cryostat with low profile blades. For immunostaining, bone sections were rehydrated in PBS, permeabilized for about 15 min in 0.5% Triton X100 PBS solution and blocked for about 30 min in PBS with 1% BSA, 2% donkey serum, 0.3% Trion X100 PBS (blocking buffer) at room temperature. Sections were probed with primary antibodies diluted in blocking buffer at 4 °C overnight. After incubation, sections were washed three times with PBS and incubated with appropriate secondary antibodies diluted in blocking buffer at room temperature for 2 h. Nuclei were stained with DAPI during secondary antibody incubation. After that, the sections were washed for three times with PBS, mounted with Fluoromount-G (0100–01, Southern Biotech) and kept in 4 °C for confocal imaging. The primary antibodies CD38 (Biolegend, 356,608), c-Kit (R&D, AF1356) and CD31(R&D, AF3628) were used in this study. The sections were imaged with laser scanning confocal microscopes (Leica SP5, LSM780, LSM800) after immunohistochemistry. We used Fiji (open source; http://fiji.sc/) for image processing in compliance with *Cell regeneration* guide for digital images.

### scRNA-seq analysis

#### Processing of sequencing data

Sequencing data from different species were aligned to the mouse reference genome (mm10) or human reference genome (hg19) and quantified using the STAR v2.7.10b software package with default parameters.

Data normalization, downstream analysis, and visualization were performed using Seurat v4.3.0 unless otherwise stated. For initial quality control of the extracted gene-cell matrices, cells were filtered based on the following criteria: nFeature_RNA > 800 and nFeature_RNA < 5000 for the number of genes detected per cell, and percent.mt < 12 for the percentage of mitochondrial genes. Additionally, genes expressed in at least 3 cells (min.cell = 3) and detected in at least 200 features (min.features = 200) were retained for further analysis. Doublet cells were identified and removed using the Scrublet v0.2.3 software with default parameters.

#### Data integration and dimensionality reduction

To integrate datasets derived from two different stages, we utilized the FindIntegrationAnchors and IntegrateData functions from the Seurat package, following the default parameters unless otherwise specified, to minimize potential batch effects. Variable features for each condition were identified using the FindVariableFeatures function with the vst selection method, selecting the top 2000 most variable features. Common variable features across conditions were identified using the SelectIntegrationFeatures function. For the integration process, anchors were identified using FindIntegrationAnchors, specifying the previously identified 2000 variable features as anchor.features. Integration was then performed using Canonical Correlation Analysis (CCA) as the reduction method. Subsequently, the IntegrateData function was applied to integrate the datasets, utilizing the identified anchors to align them into a common latent space. After integration, the integrated assay was set as the default, and the data were scaled using the ScaleData function to center and scale the expression values. RunPCA and RunUMAP functions were used for dimensionality reduction.

For integrating datasets derived from multiple stages, we employed the Seurat integration workflow based on SCTransform. In this approach, the datasets were first normalized and variance-stabilized using the SCTransform function. To integrate datasets from multiple samples while mitigating technical batch effects, we selected 3,000 features that were consistently variable across all datasets using the SelectIntegrationFeatures function. The datasets were then prepared for integration using PrepSCTIntegration, which ensures that the correct model parameters are set for the subsequent anchor-finding step in the SCT space. Integration anchors were identified with FindIntegrationAnchors, specifying normalization.method by SCT and using the 3,000 previously identified variable features as anchor.features. Once the anchors were identified, the datasets were integrated into a single, batch-corrected matrix using the IntegrateData function, again specifying normalization.method by SCT. This function applies the identified anchors to learn a correction vector for each cell, aligning the space suitable for joint analysis.

Finally, standard dimensionality reduction and visualization techniques were applied to the integrated dataset. PCA was performed using RunPCA, with the top principal components used for subsequent nonlinear dimensionality reduction. Nonlinear dimensionality reduction was performed using RunUMAP, and cells were visualized in two dimensions using UMAP.

#### Significant gene expression and functional analysis

The cellular identity of each cluster was determined by identifying cluster-specific marker genes using the FindAllMarkers function, followed by comparison of these markers with known cell type-specific genes from previous studies. Visualization of selected genes was performed using the FeaturePlot, VlnPlot, DotPlot, and DoHeatmap functions from the Seurat package.

To identify differentially expressed genes (DEG) across clusters, the FindAllMarkers function was employed. Genes with a log2 fold change (log2 FC) > ± 0.5 and log2 mean expression > 0.5 were considered significant DEG. Gene ontology (GO) enrichment analysis was conducted using the clusterProfiler v4.6.0 software, with a significance threshold of *P*-value < 0.05. The results were further clustered and visualized using the aPEAR v1.0 package.

Initially, the AverageExpression function was applied to calculate the average gene expression levels across conditions. Subsequently, the data were scaled within each condition using the scale function in R. Finally, the scaled expression values were visualized using the ComplexHeatmap package. Similarly, the interaction strength between cells was scaled and visualized using the ComplexHeatmap package.

#### Module score calculation

Additional gene sets were curated from the Gene Ontology (GO) database. The AddModuleScore function in Seurat was employed to compute scores for each gene set, which were subsequently rescaled to a 0–10 scale across all sample cells. These scores were calculated and visualized using R v4.2.2 and the ggplot2 v3.4.0 package.

#### Correlation analysis

To assess the similarity between different conditions, Pearson correlation analysis was performed based on differentially expressed genes (DEG) identified across groups. The significance of each gene within each group was evaluated. The top 3,000 most variable genes across groups were selected for correlation analysis, which was conducted using the cor function in R with default parameters. The results were visualized using the ggplot2 v3.4.0 package.

#### Cell–cell communication analysis

Cell–cell interaction analysis was conducted using the CellChat v1.6.1 software to predict ligand-receptor interactions and infer cellular communication networks. Default parameters were applied throughout the analysis. The interactions between cell types in each condition were visualized using bar plots, dot plots, heatmaps, and river plots, generated with the ggplot2 v3.4.0 and ggalt v0.1.1 packages. Additionally, ligand-receptor interactions were further visualized using the circlize v0.4.15 and iTALK v0.1.0 packages.

To identify common and unique ligand-receptor pairs across different conditions, the upsetR v1.4.0 and ggplot2 v3.4.0 packages were employed for visualization. For the analysis of human-mouse embryo-adult common cell interactions, the Cytoscape v3.9.1 software was utilized to generate and visualize the results.

#### Cross-species data integration

For cross-species analysis, human gene symbols were first translated into their corresponding mouse gene symbols using the human-mouse homologous gene table provided by the Ensemble genome browser. Subsequently, the SCTransform, FindIntegrationAnchors, and IntegrateData functions were employed to integrate the four datasets.

### Data and code availability

In this study, all single-cell RNA sequencing datasets were obtained from publicly accessible repositories and the hematopoietic microenvironment atlas data has been uploaded in (OMIX009972, https://ngdc.cncb.ac.cn/omix/release/OMIX009972), as detailed below. The hematopoietic microenvironment datasets included: human embryo bone marrow (available at 10.6084/m9.figshare.21253758.v1), human adult bone marrow (GSE253355), mouse embryonic liver (CRA002489), mouse embryonic bone marrow (GSE152285), mouse postnatal bone marrow (PRJNA835050, GSE128761), mouse juvenile bone marrow (GSE128761, GSE156635, GSE156636), mouse adult bone marrow (GSE118436, GSE108892), and mouse aging bone marrow (GSE169162). These datasets are publicly available (Bandyopadhyay et al. 2024; Gao et al. 2022; Kara et al. 2023; Li et al. 2020; Liu et al. 2022; Mitchell et al. 2023; Sivaraj et al. 2021; Tikhonova et al. 2019; Zheng et al. 2022) and were not originally generated as part of this study.

All analytical scripts and computational workflows employed in this study are publicly accessible through the vignettes provided on the respective software websites. Relevant sources have been appropriately cited in the Methods section. No custom code or novel mathematical algorithms, beyond standard variable assignments, were utilized in this study.

For request, please contact the lead contact, Qi Chen (chen_qi@gibh.ac.cn).

## Supplementary Information

Supplementary Material 1: Fig. S1. Developmental differences of human embryonic and adult BM microenvironment. Fig. S2. Comparison of human and mice BM microenvironment across developmental stages. Fig. S3. Developmental dynamics of mouse BM microenvironment. Fig. S4. Comparison of liver and BM vascular niche in embryonic stage. Fig. S5. Aging remodels the transcriptome of BM microenvironment. Fig. S6. Midkine knockout mice exhibits normal hematopoiesis in adult bone marrow.

Supplementary Material 2: Table S1. Description of all data sources in the hematopoietic microenvironment atlas.

## Data Availability

Data in this paper are openly available.

## Abbreviations

HMA: Hematopoietic microenvironment atlas
VEC: Vascular endothelial cell
SC: Stromal cell
HSPC: Hematopoietic stem and progenitor cell
BM: Bone marrow
BMEC: Bone marrow endothelial cells
BMSC: Bone marrow mesenchymal stromal cells
OLC: Osteolineage cells
LSK: Lin⁻Sca1⁺cKit⁺
HUVECs: Human umbilical vein endothelial cells
scRNA-seq: Single-cell RNA sequencing
GO: Gene ontology
DEG: Differentially-expressed genes
PCA: Principal Component Analysis
UMAP: Uniform Manifold Approximation and Projection
FACS: Fluorescence-Activated Cell Sorting
EdU: 5-Ethynyl-2'-Deoxyuridine
CFU: Colony forming unit
MDK: Midkine
iMDK: Midkine inhibitor
EGCS: Endothelial Cell Growth Supplement
