# Supplementary material for: A single-cell hematopoietic microenvironmental atlas reveals progressive maturation of bone marrow vascular niche
Source: Cell Regen. 2025 Dec 4;14:50. doi: 10.1186/s13619-025-00265-7 (PMC12675905; doi:10.1186/s13619-025-00265-7)
Supplement: Supplementary file 1 — Supplementary Material 1: Fig. S1. Developmental differences of human embryonic and adult BM microenvironment. Fig. S2. Comparison of human and mice BM microenvironment across developmental stages. Fig. S3. Developmental dynamics of mouse BM microenvironment. Fig. S4. Comparison of liver and BM vascular niche in embryonic stage. Fig. S5. Aging remodels the transcriptome of BM microenvironment. Fig. S6. Midkine knockout mice exhibits normal hematopoiesis in adult bone marrow. [file 13619_2025_265_MOESM1_ESM.pdf]

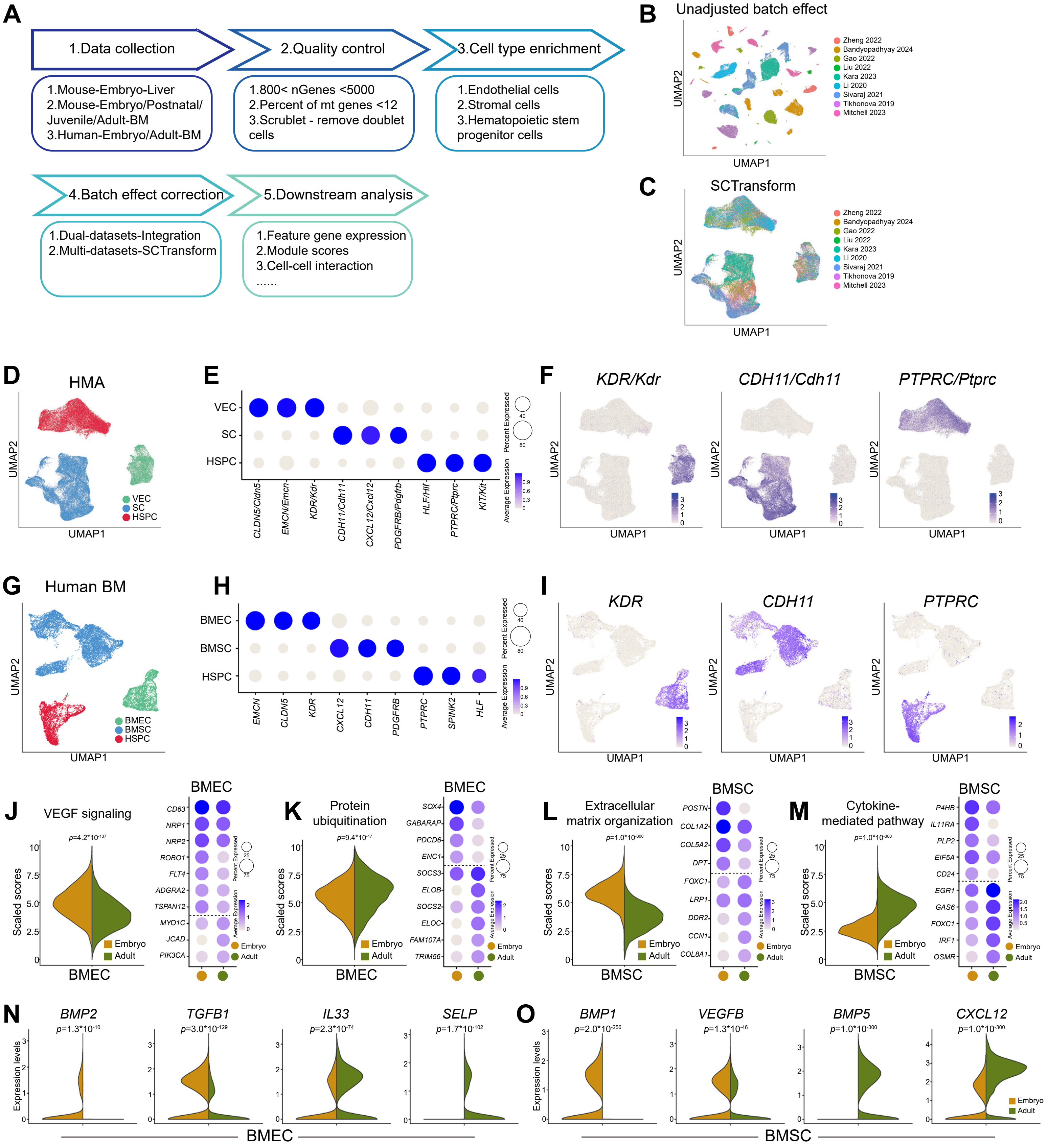

Ma et al, Figure S01

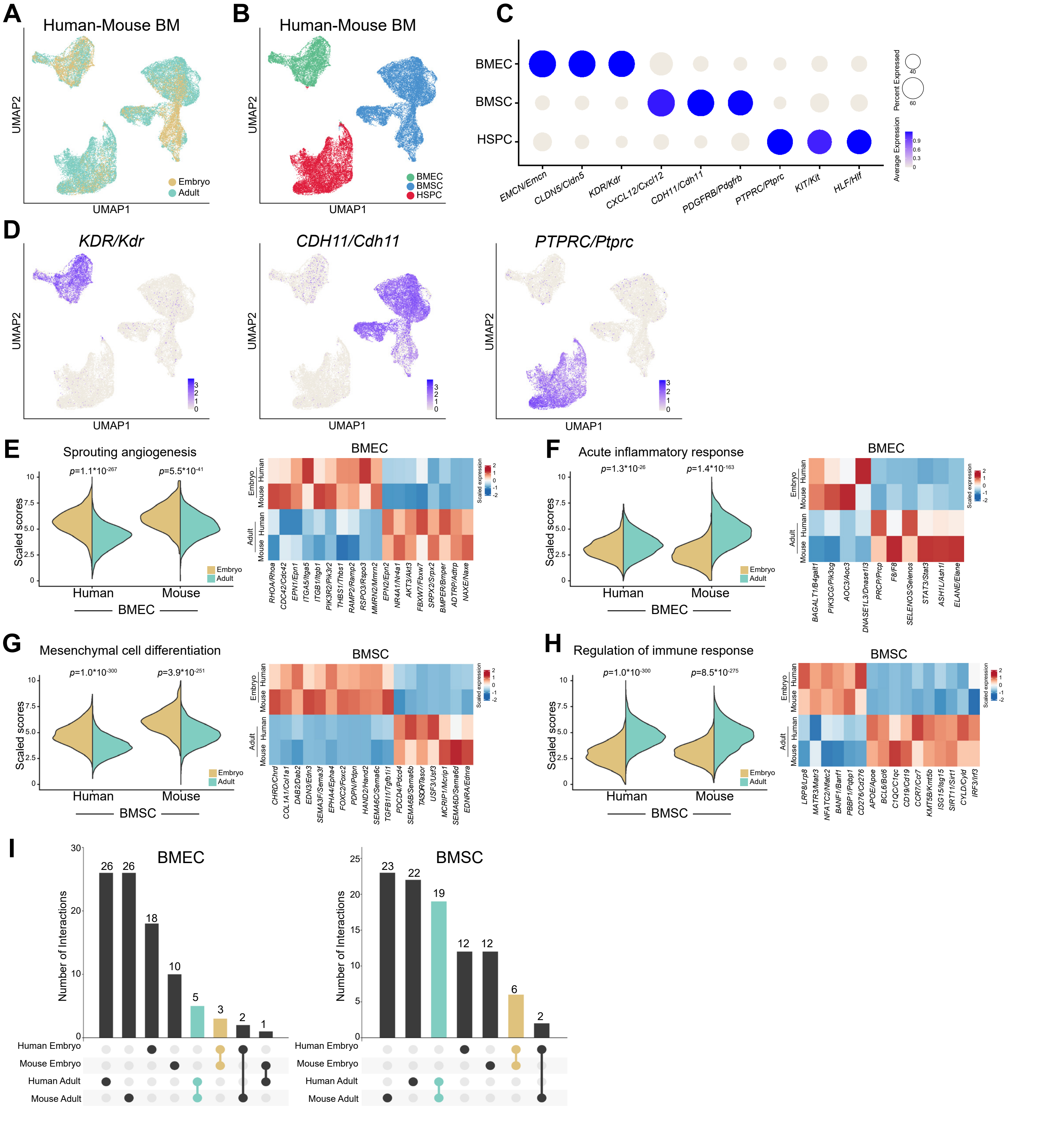

Ma et al, Figure S02

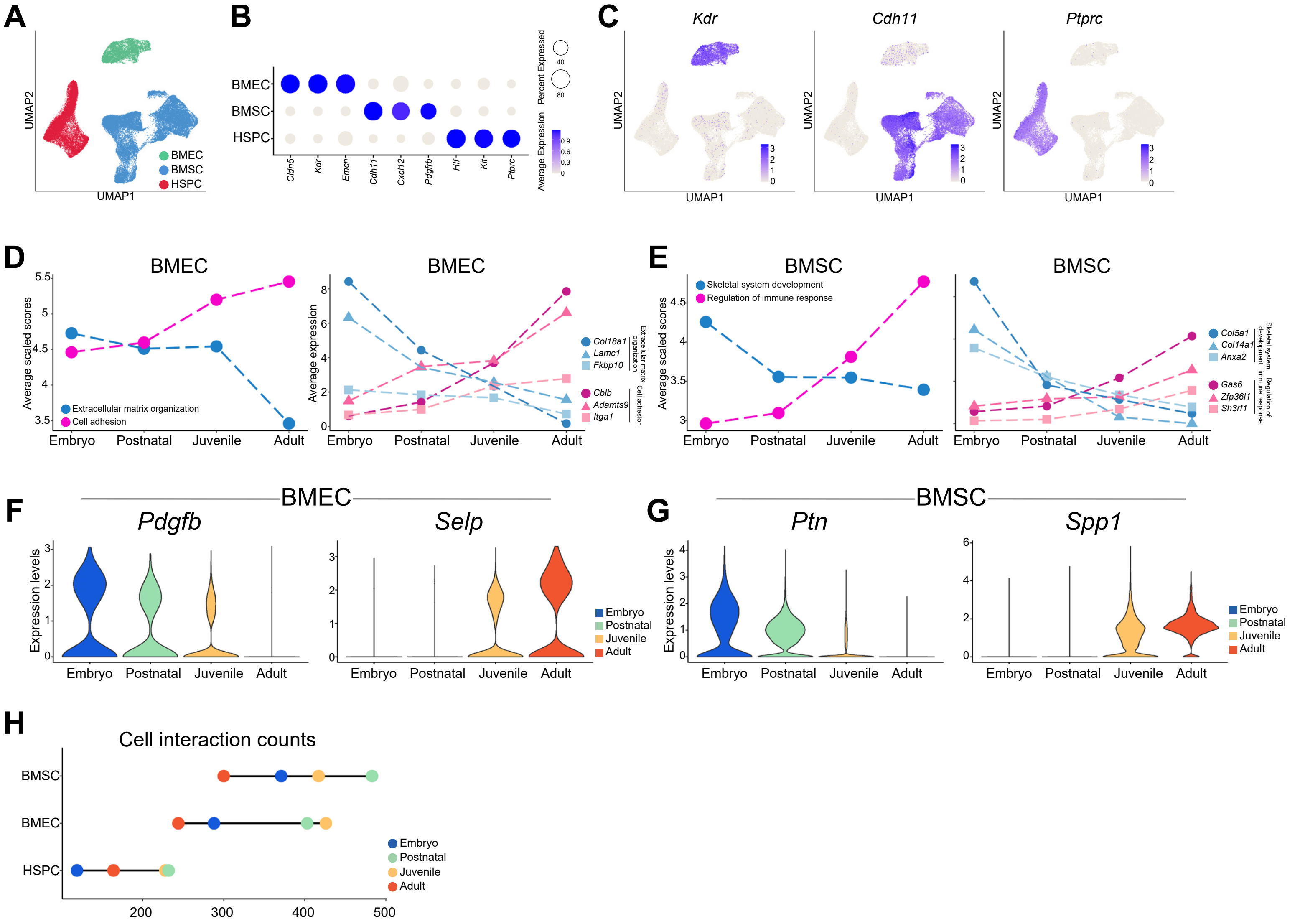

Ma et al, Figure S03

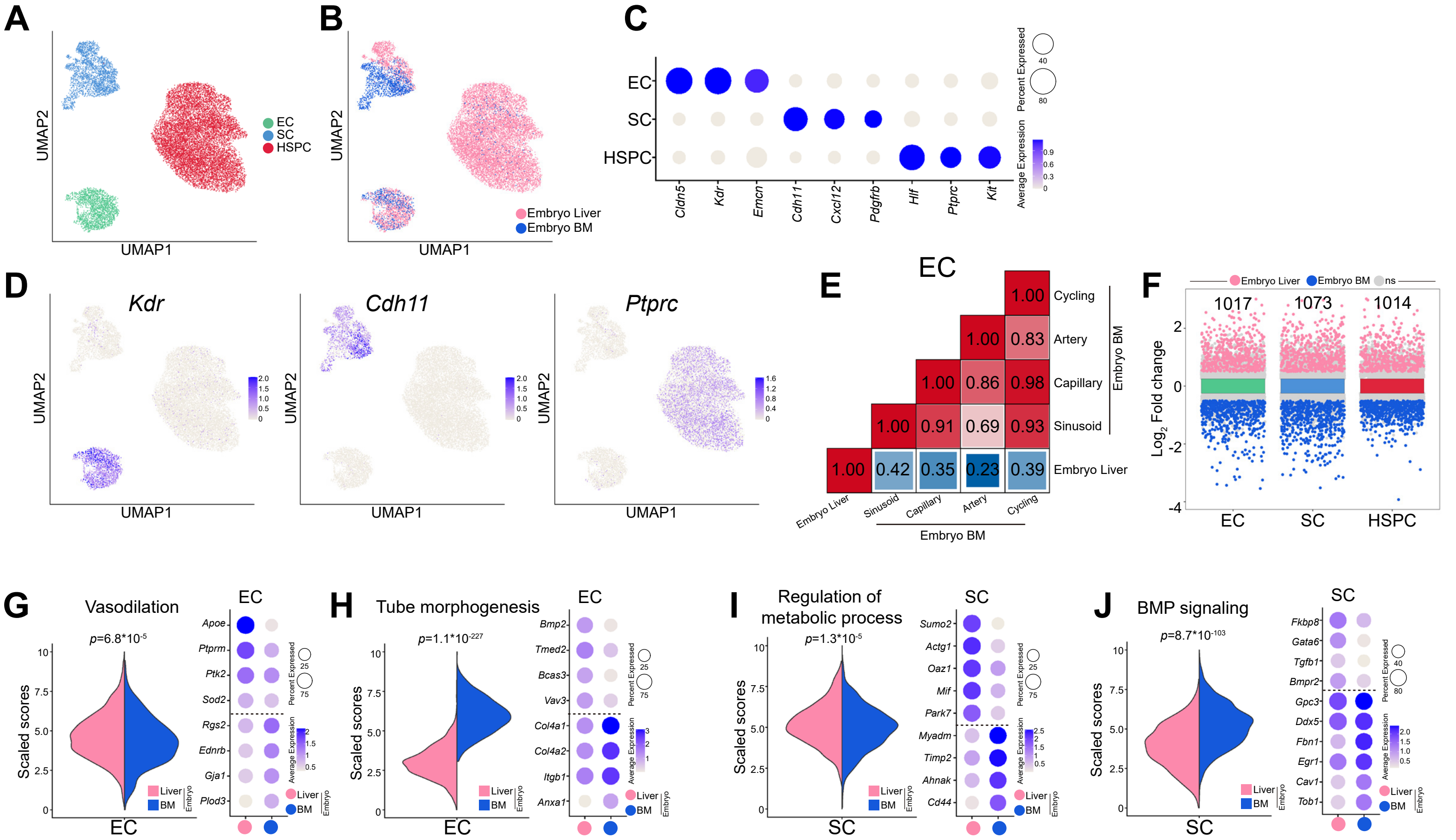

Ma et al, Figure S04

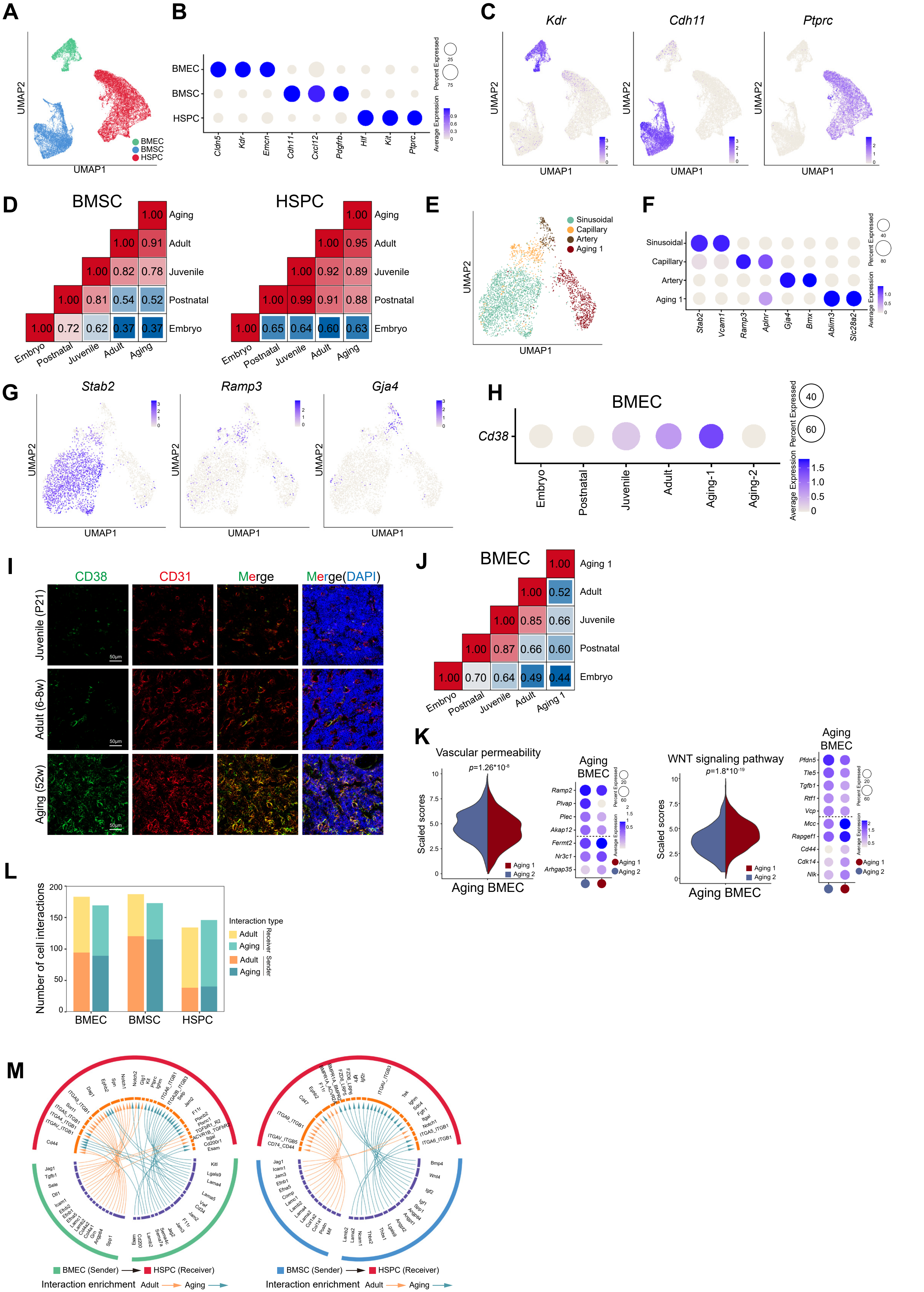

Ma et al, Figure S05

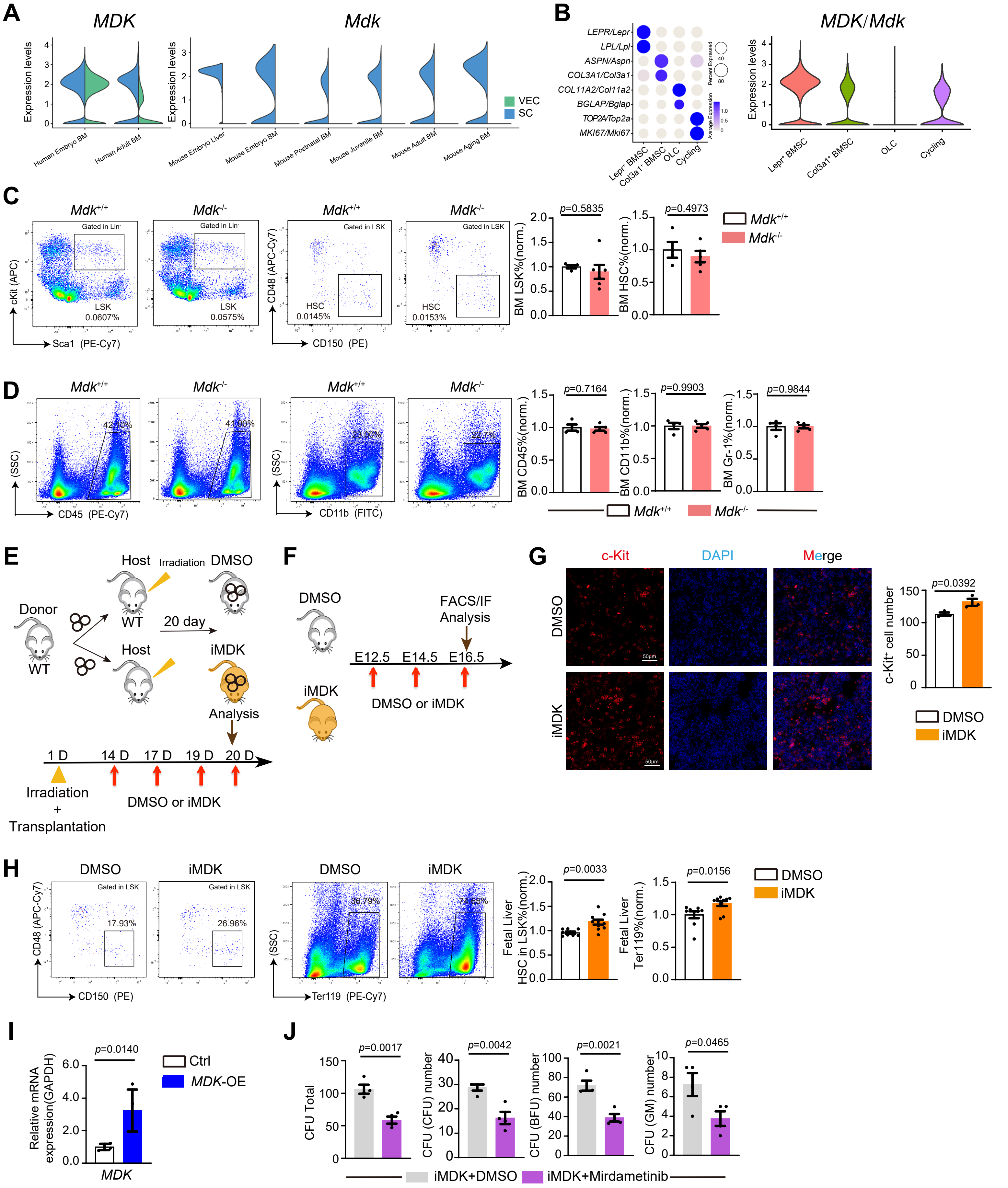
A
MDK
Mdk
Expression levels
VEC
SC
Human Embryo BM
Human Adult BM
Mouse Embryo Liver
Mouse Embryo BM
Mouse Postnatal BM
Mouse Juvenile BM
Mouse Adult BM
Mouse Aging BM
B
LEPR/Lepr
LPL/Lpl
ASPN/Aspn
COL3A1/Col3a1
COL11A2/Col11a2
BGLAP/Bglap
TOP2A/Top2a
MKI67/Mki67
Percent Expressed
Average Expression
Lepr+ BMSC
Col3a1+ BMSC
OLC
Cycling
MDK/Mdk
C
Mdk+/+
Mdk-/-
Gated in Lin-
Gated in LSK
LSK 0.0607%
LSK 0.0575%
HSC 0.0145%
HSC 0.0153%
cKit (APC)
Sca1 (PE-Cy7)
CD48 (APC-Cy7)
CD150 (PE)
p=0.5835
p=0.4973
BM LSK%(norm.)
BM HSC%(norm.)
D
42.10%
41.90%
24.00%
22.7%
(SSC)
CD45 (PE-Cy7)
CD11b (FITC)
p=0.7164
p=0.9903
p=0.9844
BM CD45%(norm.)
BM CD11b%(norm.)
BM Gr-1%(norm.)
E
Host
Irradiation
DMSO
Donor
WT
20 day
iMDK
Analysis
1 D
14 D
17 D
19 D
20 D
Irradiation
+
Transplantation
DMSO or iMDK
F
DMSO
iMDK
FACS/IF
Analysis
E12.5
E14.5
E16.5
DMSO or iMDK
G
c-Kit
DAPI
Merge
DMSO
iMDK
50μm
p=0.0392
c-Kit+ cell number
H
DMSO
iMDK
Gated in LSK
17.93%
26.96%
36.79%
74.65%
CD48 (APC-Cy7)
CD150 (PE)
(SSC)
Ter119 (PE-Cy7)
p=0.0033
p=0.0156
Fetal Liver HSC in LSK%(norm.)
Fetal Liver Ter119%(norm.)
I
p=0.0140
Relative mRNA expression(GAPDH)
Ctrl
MDK-OE
MDK
J
p=0.0017
p=0.0042
p=0.0021
p=0.0465
CFU Total
CFU (CFU) number
CFU (BFU) number
CFU (GM) number
iMDK+DMSO
iMDK+Mirdametinib

Supplementary Figure Legends

Fig. S1. Developmental differences of human embryonic and adult BM microenvironment.

(A) Schematic diagram outlining the workflow for constructing the HMA.

(B-C) UMAP plot illustrating the effects of SCTransform integration on the HMA.

(D) UMAP plot showing different cell types in HMA.

(E) Dot plot showing the representative molecular markers that were used to distinguish different cell types in HMA.

(F) UMAP plot showing typical markers (*KDR*/*Kdr*, *CDH11*/*Cdh11*, *PTPRC*/*Ptprc*) with different cell types in HMA.

(G) UMAP plot showing different cell types in human BM with embryonic and adult.

(H) Dot plot showing the representative molecular markers that were used to distinguish different cell types in human BM with embryonic and adult.

(I) UMAP plot showing typical markers (*KDR*, *CDH11*, *PTPRC*) with different cell types in human BM with embryonic and adult.

(J-K) Violin plot showing VEGF signaling and protein ubiquitination scores and dot plot showing genes associated with human embryonic and adult BMEC.

(L-M) Violin plot showing extracellular matrix organization and cytokine-mediated pathway scores and dot plot showing genes associated with human embryonic and adult BMSC.

(N-O) Volin plot of niche factors (*BMP2*, *TGFB1*, *IL33*, *SELP*, *BMP1*, *VEGFB*, *BMP5*, *CXLC12*) with embryo and adult in human BMEC or BMSC.

Fig. S2. Comparison of human and mice BM microenvironment across developmental stages.

(A) UMAP plot showing different developmental stages in human and mouse BM.

(B) UMAP plot showing different cell types in human and mouse BM.

(C) Dot plot showing the representative molecular markers that were used to distinguish different cell types in human and mouse BM.

(D) UMAP plot showing typical markers (*KDR*/*Kdr*, *CDH11*/*Cdh11*, *PTPRC*/*Ptprc*) with different cell types in human and mouse BM.

(E-F) Violin plots of sprouting angiogenesis and acute inflammatory response scores and heatmap of cross-species conserved and stage-specific genes in BMEC.

(G-H) Violin plots of mesenchymal cell differentiation and regulation of immune response scores and heatmap of cross-species conserved and stage-specific genes in BMSC.

(I) Upset plot showing evolutionarily conserved and stage-enriched HSPC as receiver interactions mediated by BMEC or BMSC as senders across species.

Fig. S3. Developmental dynamics of mouse BM microenvironment.

(A) UMAP plot showing different cell types in mouse developmental BM.

(B) Dot plot showing the representative molecular markers that were used to distinguish different cell types in mouse developmental BM.

(C) UMAP plot showing typical markers (*Kdr*, *Cdh11*, *Ptprc*) with different cell types in mouse developmental BM.

(D) Developmental dynamics of extracellular matrix organization and cell adhesion scores and associated gene expression in mouse developmental BMEC.

(E) Developmental dynamics of skeletal system development and regulation of immune response scores and associated gene expression in mouse developmental BMSC.

(F-G) Violin plot showing niche factors expression (*Pdgfb*, *Selp*, *Ptn*, *Spp1*) in mouse developmental BMEC or BMSC.

(H) Number of different cell types in cell-cell interactions in mouse developmental BM.

Fig. S4. Comparison of liver and BM vascular niche in embryonic stage.

(A) UMAP plot showing different tissues in embryonic liver and BM.

(B) UMAP plot showing different tissues in embryonic hematopoietic microenvironment.

(C) Dot plot showing the representative molecular markers that were used to distinguish different cell types in embryonic liver and BM.

(D) UMAP plot showing typical markers (*Kdr*, *Cdh11*, *Ptprc*) with different cell types in embryonic liver and BM.

(E) Heatmap of correlation between embryonic liver and BM subclusters in EC.

(F) Quantitative comparison of DEG counts between embryonic liver and BM.

(G-H) Violin plot showing vasodilation and tube morphogenesis scores and dot plot showing genes associated with embryonic liver and BM in EC.

(I-J) Violin plot showing regulation of metabolic process and BMP signaling scores and dot plot showing genes associated with embryonic liver and BM in SC.

Fig. S5. Aging remodels the transcriptome of BM microenvironment.

(A) UMAP plot showing different cell types in adult and aging BM.

(B) Dot plot showing the representative molecular markers that were used to distinguish different cell types in adult and aging BM.

(C) UMAP plot showing typical markers (*Kdr*, *Cdh11*, *Ptprc*) with different cell types in adult and aging BM.

(D) Heatmap showing correlation of BMSC and HSPC between aging and developmental BM.

(E) UMAP plot showing subclusters of BMEC with adult and aging.

(F) Dot plot showing the representative molecular markers that were used to distinguish subclusters in adult and aging BMEC.

(G) UMAP plot showing typical markers (*Stab2, Ramp3, Gja4*) with different subclusters in adult and aging BMEC.

(H) Dot plot showing the *Cd38* expression across developmental and aging subclusters in BMEC.

(I) Immunofluorescence analysis of CD38 (Green) and CD31 (Red) expression in femur at juvenile, adult and aging (52weeks) by confocal microscopy. Blue, DAPI. Scale bar, 50μm.

(J) Heatmap showing correlation of aging 1 and developmental BM in BMEC.

(K) Violin plots showing vascular permeability and WNT signaling pathway scores and dot plot showing genes associated with aging 1 and aging 2 in aging BMEC.

(L) Number of cell-cell communication pathways in BMEC, BMSC, and HSPC in adult and aging BM.

(M) Enrichment signaling from BMEC or BMSC to HSPC in adult and aging BM

Fig. S6. Midkine knockout mice exhibits normal hematopoiesis in adult bone marrow.

(A) Violin plot showing *MDK*/*Mdk* expression of non-HSPC in the atlas.

(B) Dot plot showing the representative markers that were used to distinguish different cell subclusters in BMSC including Lepr^{+} BMSC, Col3a1^{+} BMSC, osteolineage cells (OLC) and Cycling cells and violin plot showing *MDK*/*Mdk* expression of each subclusters in BMSC.

(C) Representative FACS dot plot and quantification about the percentage of Lin^{-} c-Kit^{+} sca1^{+}(LSK cell, $Mdk^{+/+}$=4, $Mdk^{-/-}$=6), Hematopoietic stem cell (HSCs, $Mdk^{+/+}$=4, $Mdk^{-/-}$=5). Error bars, mean ± SEM. p values, t- test.

(D) Representative FACS dot plot of CD45 and CD11b.Quantification about the percentage of CD45, CD11b and Gr-1($Mdk^{+/+}$=4, $Mdk^{-/-}$=5), Error bars, mean ± SEM. p values, t- test.

(E) Diagram depicting schedule for DMSO or iMDK treated mouse after lethal irradiation and transplantation as well as corresponding analyzing time point.

(F) Diagram depicting schedule for pregnant female mice treated by DMSO or iMDK at different time points.

(G) Immunofluorescence analysis of c-Kit (Red) expression in fetal liver (E16.5) that were treated with DMSO or iMDK by confocal microscopy. Quantification about the cell number of c-Kit^{+} (DMSO=3, iMDK=3). Blue, DAPI, Scale bar, 50μm. Error bars, mean ± SEM. p values, t- test.

(H) Representative FACS dot plot of LSK and Ter119.Quantification about the percentage of LSK (DMSO=8, iMDK=10) and Ter119 (DMSO=9, iMDK=10). Error bars, mean ± SEM. p values, t- test.

(I) Quantitative qRT-PCR analysis validate up-regulation of transcription of *MDK* (Ctrl=4, *MDK*-OE=4)

(J) Quantification of CFU total, CFU number, BFU or CFU -GM number derived from 20000 Lin$^-$ cell isolated from WT mice. DMSO+iMDK or iMDK+Mirdametinib was added to MethoCult medium (DMSO+iMDK=4, iMDK+Mirdametinib =4). Error bars, mean ± SEM. p values, t- test.
